# Intermittent Fasting and Risk of Diabetic Retinopathy: Retrospective Data from the National Health and Nutrition Examination Survey

**DOI:** 10.3390/nu18111696

**Published:** 2026-05-26

**Authors:** Sejeong Lee, Youngjoon Kim, Min Heui Yu, Yong-ho Lee, Byung-Wan Lee, Eun Seok Kang, Bong-Soo Cha, Soo-Hyun Park, Sungha Park, Min Kim, Christopher Seungkyu Lee, Eun Young Choi, Minyoung Lee

**Affiliations:** 1 Graduate School, Yonsei University College of Medicine, Seoul 03722, Republic of Korea; dltpwjd123@naver.com; 2 Department of Endocrinology and Metabolism, Inha University School of Medicine, Incheon 22332, Republic of Korea; dudwnsdl82@naver.com; 3 Department of Internal Medicine, Yonsei University College of Medicine, 50-1 Yonsei-ro, Seodaemun-gu, Seoul 03722, Republic of Korea; 4 SENTINEL Team, Division of Endocrinology, Department of Internal Medicine, Yonsei University College of Medicine, Seoul 03722, Republic of Korea; 5 Institute of Endocrine Research, Yonsei University College of Medicine, Seoul 03722, Republic of Korea; 6 Precision Nutrition Research Group, Food Functionality Research Division, Korea Food Research Institute, Wanju-gun 55365, Jeollabuk-do, Republic of Korea; 7 Division of Cardiology, Severance Cardiovascular Hospital, Yonsei University College of Medicine, Seoul 03722, Republic of Korea; 8 Department of Ophthalmology, Institute of Vision Research, Gangnam Severance Hospital, Yonsei University College of Medicine, 211 Eonju-ro, Gangnam-gu, Seoul 06229, Republic of Korea; 9 Department of Ophthalmology, Institute of Vision Research, Severance Eye Hospital, Yonsei University College of Medicine, Seoul 03722, Republic of Korea

**Keywords:** diabetic retinopathy, fundus photography, intermittent fasting, national health and nutrition examination survey, prevention

## Abstract

**Background/Objectives:** Diabetic retinopathy (DR) is the most common microvascular complication of diabetes and a significant cause of severe visual impairment. Intermittent fasting (IF) has demonstrated metabolic benefits. We investigated the association between IF and DR risk in individuals with prediabetes and diabetes. **Methods:** This retrospective cohort study included participants of the Korean National Health and Nutrition Examination Survey 2017–2018 aged ≥40 years who were diagnosed with diabetes or prediabetes who had fundus photography and dietary pattern data. Participants were allocated to the IF (fasting for 24 h or skipping breakfast or dinner) and regular diet groups. Demographic, dietary pattern and clinical data, including DR prevalence, were compared between the groups. Multiple logistic regression assessed the association between IF and DR risk. **Results:** Of 922 participants, 831 followed a regular diet while 91 practiced IF. The participants in the IF group were significantly younger and more obese, had higher fat intake, and showed a lower prevalence of DR than those in the regular diet group (8.8% vs. 20.6%, *p* = 0.010). After adjusting for multiple covariates, including demographics, comorbidities, health behaviors, biochemical parameters, and nutritional intake profiles, IF was associated with a 70% reduced risk of DR (OR 0.30, 95% CI 0.12–0.65, *p* = 0.005). This association did not differ across subgroups (all *p* for interaction > 0.05). **Conclusions:** IF was significantly associated with reduced DR risk in this study. Further studies are needed to validate the effectiveness of IF as a dietary intervention for DR.

## 1. Introduction

The prevalence of diabetes is rising globally, with the patient population expected to reach 429 million by 2030, coupled with an increasing burden of chronic complications [1,2]. Diabetic retinopathy (DR) is the most common microvascular complication of diabetes, affecting 27% of patients with diabetes worldwide [3,4]. Additionally, DR is a predictor of other end-organ complications [5]. Despite therapeutic advancements, including the use of vascular endothelial growth factor inhibitors, DR remains the fifth most common cause of severe visual impairment [6], accounting for blindness in approximately 1.07 million people worldwide by 2020 [7]. Therefore, preventing DR in patients with prediabetes and diabetes is essential. In addition to intensive glycemic control [8], various dietary approaches have been associated with a reduced DR risk, including high fiber intake, oily fish, the Mediterranean diet, and low calorie consumption [9]. However, the association between dietary patterns, such as intermittent fasting (IF), and DR risk has not been investigated in human studies.

IF is a dietary pattern that cycles fasting periods with unrestricted eating periods [10]. IF includes easy-to-follow dietary regiments for food restriction [11]. For example, time-restricted feeding is one of the three major types of IF regimens, where food intake is limited to a specific time window each day [11]. As a therapeutic dietary strategy, IF improves metabolic profiles, slows down aging, and exhibits anti-inflammatory properties [11,12]. Particularly, IF ameliorates hyperglycemia, obesity, and dyslipidemia in individuals with type 2 diabetes [13]. Even at the prediabetic stage, IF has been shown to exert favorable metabolic and anti-inflammatory effects, including improved insulin sensitivity, enhanced β-cell function, lower blood pressure, and reduced oxidative stress [12]. These findings suggest the therapeutic potential of IF in the treatment of ocular complications of metabolic disorders, including DR [11,14]. However, despite experimental data supporting benefits of IF for DR [15], evidence from human studies confirming an association between IF and a reduced DR risk is lacking.

Based on these backgrounds, we aimed to analyze the association between IF and DR risk in individuals with prediabetes and diabetes (including both type 1 and type 2, predominantly type 2) using National Health and Nutrition Examination Survey database.

## 2. Materials and Methods

### 2.1. Data Source and Study Population

We used data from the Korean National Health and Nutrition Examination Survey (KNHANES) for the period from 2017 to 2018. This government-led, population-based, cross-sectional survey is conducted to assess the health and nutritional status of Koreans and provides evidence of health policy. The KNHANES includes questionnaires on sociodemographic information, health-related behaviors, dietary patterns, medical history including prevalent ophthalmic diseases, biochemical profiles, and ophthalmological examination results, including fundus photography [16]. KNHANES data are publicly available. The study protocol was reviewed and approved by the Institutional Review Board of Yonsei University College of Medicine (No.4-2020-1234) and informed consent was obtained from all participants before they participated in the survey. The investigations have been carried out in accordance with the principles of the Declaration of Helsinki as revised in 2008.

We selected the 2017–2018 period because both the nutrition survey and fundus photography were conducted within this 2-year period. From a total of 16,119 participants who took part in the KNHANES 2017–2018 (*n* = 8127 in 2017; *n* = 7992 in 2018), 4374 participants who met the diagnostic criteria for diabetes and prediabetes were included. Diabetes was defined by a fasting blood glucose ≥ 126 mg/dL, glycated hemoglobin (HbA1c) ≥ 6.5%, a diagnostic history of diabetes, or using any oral or injectable antidiabetic drugs. Prediabetes was included in this study, as DR is known to begin its development even during the early dysglycemia process [17]. Prediabetes was defined as fasting blood glucose ≥ 100 mg/dL and <126 mg/dL, or HbA1c ≥ 5.7% and <6.5%. Participants were excluded if data on ophthalmic examinations for DR or meal frequency were unavailable or if they had a self-reported history of DR.

**Figure 1:**
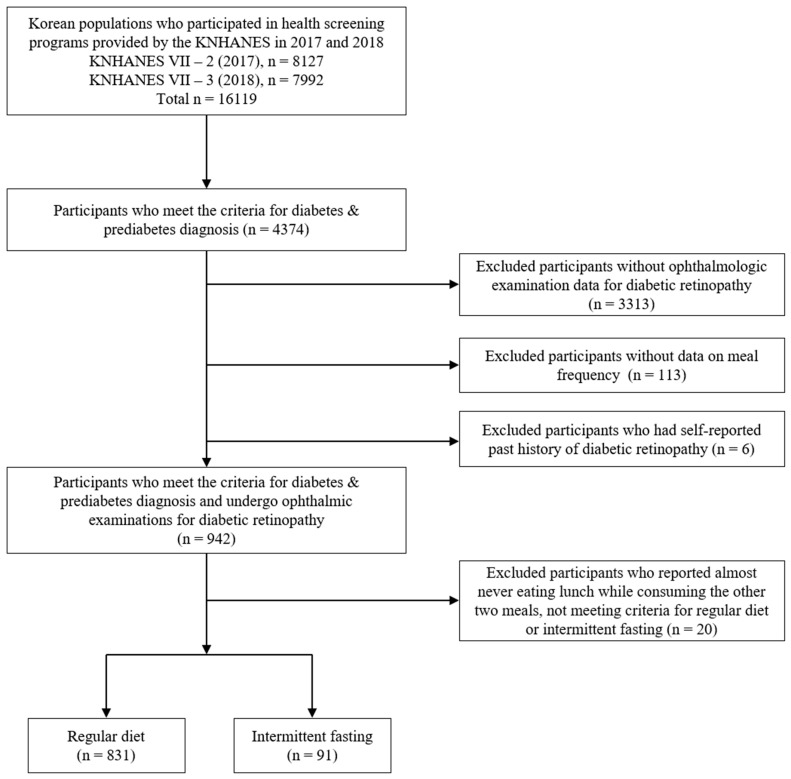
Flow chart for study subject selection.

In this study, we specifically investigated IF in the form of time-restricted feeding. We divided the study participants into IF and regular diet groups based on the responses to the following dietary interview questions: “As a method for weight control, have you skipped meals or fasted more than 24 h in the past year?” and “How many days a week have you had breakfast, lunch, and dinner in the past year?” Participants who indicated that they controlled their weight by 24 h fasting and those who regularly skipped either breakfast or dinner were considered to practice IF through time-restricted feeding (8–12 h feeding, 12–16 h fasting) [18]. This classification was based on the general observation that the time interval between dinner on one day and lunch on the next day or between lunch on one day and breakfast on the next day typically exceeds 12 h [19]. Participants who responded that they almost never eat lunch while consuming breakfast and dinner were excluded from the study, because in such cases the fasting period from after breakfast until before dinner was estimated to be approximately 10 h [19], which does not satisfy the ≥12 h fasting criterion for time-restricted feeding. These individuals were categorized as “excluded participants not meeting criteria for regular diet or IF.” The remaining participants were allocated to the regular diet group as the control group. Figure 1 summarizes a flow chart of the selection process.

### 2.2. Fundus Evaluation

In the KNHANES 2017–2018, fundus photographs were taken with a 45-degree field of view, centered on the macula, for all participants aged 40 and older [20]. The images were captured without using pupil-dilating eye drops. Instead, participants sat in a dark room for 5 min to allow natural pupil dilation. Trained ophthalmic technicians took the photos, and if an image was unclear or of poor quality, a second attempt was made. DR was diagnosed if any characteristic lesion was observed, as defined by the Early Treatment Diabetic Retinopathy Study severity scale [21]. Characteristics lesions included hemorrhage, microaneurysm, cotton wool spots, hard exudate, intraretinal microvascular abnormalities, venous beading, and neovascularization. Each fundus image was graded twice. Initially, a preliminary grading was conducted onsite by ophthalmologists or ophthalmologic residents trained by the Korean Ophthalmologic Society. Subsequently, retinal specialists with expertise performed the final grading [20]. Based on the fundus image grading, participants were divided into normal or DR groups, without further subclassifying the stage or severity of non-proliferative or proliferative DR. To identify DR diagnosed at the time of the survey, we excluded participants who had a prior diagnosis of DR, even if DR was confirmed on fundus photographs obtained during the survey.

### 2.3. Nutrition Survey

The nutrition survey in the KNHANES 2017–2018 was conducted through in-person interviews by trained medical staff, encompassing dietary behaviors, feeding frequency, and food intake. Meal skipping was addressed in the dietary behavior questionnaire. Participants provided details on all foods and beverages they consumed over a 24 h period, including a description, quantity, and the time and location of consumption for each main meal and any additional eating occasions [22]. Based on the results of the 24 h dietary recall, the daily consumption of energy and nutrients was calculated by multiplying the reported quantities of all foods and dishes by their nutrient values obtained from the nutrient database [22].

### 2.4. Outcome and Covariates

The primary outcome was the prevalence of newly developed DR. To estimate the risk for incident DR, we included only participants with newly diagnosed DR during the ophthalmic examination in KNHANES who had no DR history. We adjusted for the following covariates in the regression: age, sex, body mass index (BMI), hypertension, diabetes duration, diabetes status (diabetes vs. prediabetes), smoking, insulin use, fasting blood glucose, HbA1c, triglyceride, low-density lipoprotein (LDL)-cholesterol, alanine aminotransferase (ALT), total calories intake per day, and proportions of macronutrients including carbohydrate, protein, and fat. Hypertension was defined as a blood pressure ≥ 140/90 mmHg or self-reported treatment for hypertension [23]. Smoking was defined as having consumed over 100 cigarettes in one’s lifetime [24]. High-risk drinking was defined as drinking over 7 cups for men and over 5 cups for women per day [25]. For participants with diabetes, disease duration was calculated based on current age and age at diagnosis. However, for individuals with prediabetes, information on the exact onset was not available, making it impossible to accurately estimate disease duration. Thus, the duration of prediabetes was not defined; instead, diabetes duration was set to 0 years for participants with prediabetes.

### 2.5. Statistical Analysis

The normality of continuous variables was assessed using the Shapiro–Wilk test. Variables not meeting normality assumptions were compared using the Mann–Whitney U test. Differences in categorical variables were evaluated using the chi-square test. To assess the association between IF and DR risk, we employed multiple logistic regression analyses and estimated the odds ratio (OR) of DR with adjustment for multiple covariates.

We conducted subgroup analyses to determine whether the effect of IF remained consistent across various clinical situations. Heterogeneity in the association of IF with DR across subgroups was examined using a multiplicative interaction term. Considering the potential influence of the differences in diabetes duration between the groups on the outcome, we conducted a sensitivity analysis, specifically limiting the subjects to those with a duration of diabetes ≤ 3 or ≤5 years. In addition, to address potential imbalance in baseline characteristics between the IF and regular diet groups, we conducted a propensity score–matched (PSM) analysis. Propensity scores were estimated using clinical variables (age, sex, BMI, smoking status, drinking status, hypertension, dyslipidemia, stroke, cardiovascular disease, status of diabetes, insulin use, fasting glucose, HbA1c, total cholesterol, triglyceride, high-density lipoprotein (HDL)-cholesterol, LDL-cholesterol, blood urea nitrogen, creatinine, aspartate aminotransferase (AST), ALT, urine ketone, carbohydrate % of calorie, protein % of calorie, and fat % of calorie). A 1:1 nearest-neighbor matching approach without replacement was performed. After matching, 86 participants in the IF group were successfully matched with 86 participants in the regular diet group. The matched cohort was used to reassess the association between IF and newly developed DR. All statistical analyses were conducted using IBM SPSS Statistics version 22.0 (IBM Corp., Armonk, NY, USA) and R software version 3.6.3 (R Project for Statistical Computing, Vienna, Austria). *p* < 0.05 indicated statistical significance.

## 3. Results

### 3.1. Baseline Characteristics

The baseline characteristics are shown in Table 1. Participants in the IF group were younger and more likely to have hypertension than those in the regular diet group. Furthermore, compared with those in the regular diet group, participants in the IF group had a higher BMI, shorter diabetes duration, and worse lipid profile. When comparing total daily calorie intake and macronutrient proportions, the total calorie intake tended to be lower in the IF group than in the regular diet group; the difference was not statistically significant. The IF group had a significantly lower proportion of carbohydrate intake (64.3% vs. 69.5%, *p* = 0.002) and higher proportion of fat intake (15.9% vs. 13.9%, *p* = 0.003) than the regular diet group. The prevalence of DR was significantly lower in the IF group than in the regular diet group (8.8% vs. 20.6%, *p* = 0.010).

### 3.2. Association Between IF and DR Risk

**Figure 2:**
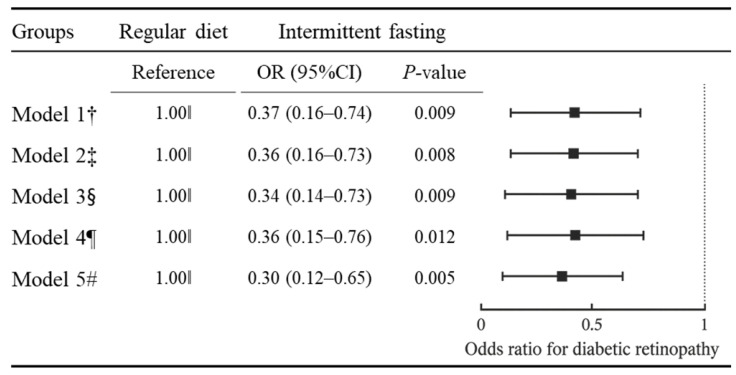
Risk of diabetic retinopathy according to intermittent fasting and regular diet. CI, confidence interval; OR, odds ratio. Model 1†: Unadjusted. Model 2‡: Adjusted for age, sex, and BMI. Model 3§: Adjusted for model 2 covariates plus hypertension, duration of diabetes, diabetes status (diabetes vs. prediabetes), and smoking. Model 4¶: Adjusted for model 3 covariates plus insulin use, fasting glucose, HbA1c, triglyceride, LDL-cholesterol, and ALT. Model 5#: Adjusted for model 4 covariates plus total calories intake per day and proportions of macronutrients; carbohydrate, protein, and fat. ǁ Regular diet group as a reference category.

**Figure 3:**
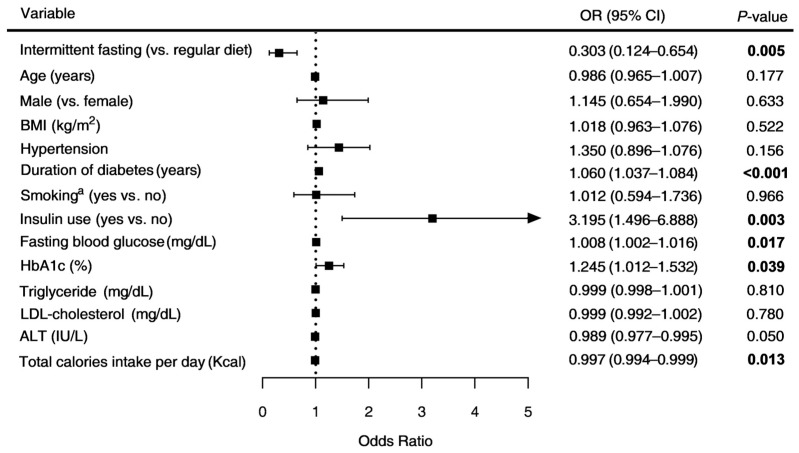
Forest plot presenting the multivariable logistic regression analysis for diabetic retinopathy. ALT, alanine aminotransferase; BMI, body mass index; CI, confidence interval; HbA1c, glycated hemoglobin; LDL, low-density lipoprotein; OR, odds ratio. ^a^ Smoking is defined as smoking over 100 cigarettes in his or her life. Model was adjusted for age, sex, BMI, hypertension, duration of diabetes, diabetes vs. prediabetes, smoking, insulin use, fasting glucose, HbA1c, triglyceride, LDL-cholesterol, ALT, total calories intake per day, and proportions of macronutrients; carbohydrate, protein, and fat. Bold font in *p*-value indicates statistical significance.

Before adjustment for covariates, IF was significantly associated with a reduced DR risk (OR 0.37, 95% confidence interval [CI] 0.16–0.74, *p* = 0.009; model 1, Figure 2). The risk reduction remained significant after adjusting for age, sex, BMI, hypertension, diabetes duration, diabetes status, smoking, insulin use, and glycemic and lipid parameters (models 2, 3, and 4; Figure 2). Furthermore, the association between IF and the reduced DR risk remained consistent and retained similar strength even after further adjustment for total daily calorie intake and the proportions of macronutrients, including carbohydrate, protein, and fat. (OR 0.30, 95% CI 0.12–0.65, *p* = 0.005, model 5; Figure 2). Conversely, longer diabetes duration, insulin use, and worse glycemic control, as evidenced by higher fasting glucose and HbA1c levels, were significantly associated with an increased DR risk (Figure 3).

To further address the potential imbalance in baseline characteristics between the IF and regular diet groups, we performed PSM analysis. In the 1:1 matched cohort, 86 participants in each group (total *n* = 172) were successfully matched and baseline characteristics were well balanced (Supplementary Table S1). Notably, the results from the PSM cohort were consistent with those of the primary analysis. In multiple logistic regression models using the matched sample, the association between IF and DR remained stable after sequential adjustment for demographic, clinical, dietary, and biochemical variables (Supplementary Table S2). In the fully adjusted PSM model incorporating diabetes duration, IF remained independently associated with a reduced risk of DR (OR, 0.135; 95% CI, 0.034–0.430; *p* = 0.001; Supplementary Table S3).

### 3.3. Subgroup and Sensitivity Analyses

The results of subgroup analyses showed that the reduced risk of DR associated with IF versus regular diet was consistent across subgroups of age, sex, BMI (<23 and ≥23 kg/m^2^), hypertension, diabetes duration, smoking status, insulin use, metabolic parameters (fasting glucose, HbA1c, triglyceride, LDL-cholesterol, and ALT), overeating, and the proportions of each macronutrient without significant interaction (all *p* > 0.05; Supplementary Table S4). This suggests that IF was significantly associated with a reduced risk of DR irrespective of interactions with other clinical variables.

Given that diabetes duration may influence DR development and progression [26], we conducted a further sensitivity analysis according to diabetes duration (Supplementary Table S5). In participants with duration of diabetes ≤ 3 years, the disease duration was comparable between the IF and regular diet groups (0.0 [0.0–12.0] vs. 0.0 [0.0–12.0] months, *p* = 0.882). In this subpopulation, IF was associated with a significantly lowered risk for DR (OR 0.26, 95% CI 0.07–0.77, *p* = 0.027). Similarly, in participants who had diabetes duration ≤ 5 years, no difference was observed in the disease duration between the groups (0.0 [0.0–18.0] vs. 0.0 [0.0–24.0] months, *p* = 0.184). The results were also generally consistent with those from the main analysis (OR 0.30, 95% CI 0.08–0.83, *p* = 0.037). Taken together, IF was significantly associated with a reduced DR risk across sensitivity analyses based on the duration of diabetes.

## 4. Discussion

A high prevalence of DR persists among individuals with diabetes, even with optimal management of blood glucose, blood pressure, lipids, and the use of anti-vascular growth factor agents [27]. Dietary interventions present promising treatment options for ocular disorders including DR, given their ease of acceptance and implementation [11]. Several animal studies have suggested favorable results of IF in DR [4,11,15]. However, to the best of our knowledge, no report has previously demonstrated these effects using clinical data. Herein, using data of individuals with prediabetes and diabetes from a National Health and Nutrition Examination Survey, we identified a 70% reduction in the risk for incident DR in individuals practicing IF. All tests for interaction across subgroups stratified by demographic, clinical, dietary, and biochemical factors yielded *p*-values greater than 0.05, indicating no statistically significant effect modification. The risk reduction associated with IF was generally consistent in the sensitivity analysis, with respect to diabetes duration. Even in the 1:1 PSM cohort, IF remained associated with a reduced risk of DR.

Energy restriction is an important therapeutic approach leading to improvement of glucose control in patients with diabetes. IF is physiological way to prevent the harmful effects of chronic excess of food intake. IF encompasses a variety of approaches, including time-restricted feeding, alternate-day fasting, 5:2 regimen, one-meal-a-day, and periodic whole-day fasting. Recent meta-analyses have shown that all forms of IF can improve metabolic health, with alternate-day fasting demonstrating the most favorable outcomes [28,29]. However, time-restricted feeding represents the simplest and most sustainable form of IF, making it the major subtype and potentially the most representative approach [18]. Because our analysis was limited to dietary patterns identifiable from the KNHANES questionnaire, our findings are restricted to the impact of time-restricted feeding on DR, and we were not able to evaluate other IF regimens. IF by time-restricted feeding resulted in greater weight loss, greater improvement in glycemic control, β-cell function, and insulin sensitivity, and a more significant decrease in atherosclerotic lipid levels than the control normal diet in patients with type 2 diabetes [13]. Time-restricted feeding showed positive results in patients with type 2 diabetes and those with prediabetes and metabolic syndrome, improving cardiometabolic profiles, including adiposity, blood pressure, and glycemic parameters [12,30]. Thus, IF may contribute to lowering the risk of diabetic complications by modifying the cardiometabolic risk factors [31]. Furthermore, the metabolic benefits of IF in both individuals with diabetes and those at high risk for developing diabetes suggest that IF could positively affect DR, one of the earliest complications of diabetes, even developed in prediabetic stages [17]. However, establishing the positive effects of IF on the prognosis of diabetic complications, including DR, in prospective clinical studies is challenging because it requires a large number of participants to adhere to the IF diet over a long period. To the best of our knowledge, no clinical studies have specifically investigated the effect of IF on the incidence of DR. Thus, retrospective cross-sectional studies based on well-documented data about dietary habits and nutrient intake can be a practical alternative research design for investigating the association between IF diet and the outcomes of interest. A notable strength of the present study is the use of data from national survey, which included questionnaires on dietary behavior. This enabled us to identify individuals who were following IF diet among a large number of survey participants. Additionally, by integrating the results of fundus examinations, the impact of IF dietary pattern on DR risk could be examined. Our findings therefore highlight the need for future large-scale prospective cohort studies to determine whether specific IF subtypes may individually exert favorable effects on the risk of DR.

In this cohort study, due to the detailed dietary consumption data, we could estimate total daily calorie intake and the intake proportions of macronutrients such as carbohydrates, proteins, and fats and incorporate these dietary factors into the analyses. Although IF through time-restricted feeding is a dietary regimen not involving calorie counting, it naturally tends to reduce daily energy intake [18]. Hence, some of the metabolic benefits of IF may be attributable to the reduction in total energy intake, similar to a calorie restriction diet [18]. However, even after adjustment for total daily calorie intake and intake proportions of macronutrients, the association between IF and reduced risk for DR remained consistent in this study. These findings suggest that IF may have unique mechanisms underlying its metabolic advantage beyond simply reducing daily energy intake and body weight. This is evidenced by previous reports showing that IF produced positive changes in metabolic parameters even when compared to control groups with an isocaloric diet or comparable weight loss [18,32].

Several potential mechanisms, not limited to mere calorie restriction, may explain the favorable results of IF in DR. First, ketogenic response triggered by IF may have positively affected DR. With prolonged fasting, such as that experienced in the IF diet, liver glycogen gets depleted, leading to ketone production in the liver as an alternative energy source during fasting [33]. Ketones provide potential therapeutic advantages in several clinical areas, including metabolic disorders like diabetes, exerting their anti-inflammatory properties [34,35,36]. Ketones may also modulate endothelial cell function and exert a vasodilatory effect, which could be beneficial for cardiovascular functions and blood flow [37]. These effects of ketones, including glycemic improvement, anti-inflammatory properties, and preservation of blood flow, may contribute to the amelioration of the pathophysiology of DR, where microvascular damage due to hyperglycemia is a main mechanism [4]. Second, low-nutrient states induced by IF activate sirtuin 1 (SIRT1), a nutrient-sensing deacetylase that enhances insulin secretion and attenuates insulin resistance, inflammation, and mitochondrial damage [11]. In this metabolic and anti-inflammatory context, SIRT1 can positively impact DR. At the same time, upregulated SIRT1 activity in the retina by IF may ameliorate DR by normalizing cholesterol metabolism [4]. SIRT1 activation enhances deacetylation of liver X receptor alpha and expressions of relevant genes, which results in increased cholesterol export and decreased cholesterol levels in retinal endothelial cells [4]. Third, IF-mediated changes in the gut microbiota have the potential to protect against DR [15]. In a diabetic mouse model, IF showed better outcomes in the vascular and inflammatory end points of DR, restructuring the composition of gut microbiota toward species producing tauroursodeoxycholate, a neuroprotective bile acid [15]. Tauroursodeoxycholate circulates and activates its target receptor, TGR5, in retinal neuronal cells, and the activation of TGR5 can alleviate the pathophysiology of DR by suppressing inflammatory responses [15].

In our study, the duration of diabetes was significantly shorter in the IF group compared with the regular diet group (1 vs. 3 years). Although diabetes duration is a well-established risk factor for the development and progression of DR [38], a mean difference of two years has been reported to have little clinical impact on incidence rates. For instance, data from large cohorts of patients with type 2 diabetes suggest that a 3-year screening interval may be safe for those without evidence of retinopathy [39], while a 5-year interval has been suggested for patients with type 1 diabetes [40]. Nonetheless, to minimize potential confounding, we performed multiple logistic regression adjusting for diabetes duration as well as other demographic, clinical, biochemical, and nutritional factors. Each additional year of diabetes duration was associated with a 6% increase in DR risk (OR = 1.060), yet IF remained significantly associated with a reduced risk of DR even after full adjustment (OR = 0.30). Furthermore, even in a PSM cohort designed to minimize baseline imbalances between the IF and control groups, the protective association of IF with DR risk was consistently independent of duration of diabetes. Prospective randomized controlled trials reducing imbalance in key variables, including diabetes duration, are warranted to validate the effect of IF on DR.

This study has several limitations. First, owing to the retrospective and cross-sectional design, the limited sample size, and potential residual confounding between groups, the findings should be regarded as preliminary and cannot establish causal relationships. In addition, because a memory-dependent survey methodology was used, the accuracy of the data may be limited. In particular, self-reported dietary assessments using 24 h recalls are known to be subject to both random errors, which reduce precision, and systematic errors, such as recall bias and energy underreporting, which compromise accuracy [41]. Although KNHANES applied a standardized multiple-pass method in 24 h recalls to minimize errors [42], recall bias cannot be fully eliminated. Furthermore, the questionnaire does not allow determination of the exact number of fasting episodes, the heterogeneity of fasting strategies, or the total duration of intermittent fasting adherence. Therefore, despite the widespread use of this approach in large-scale surveys due to its feasibility, these methodological limitations should be considered when interpreting the study findings. Second, the lack of detailed information on the grade and severity of DR prevented us from investigating the impact of IF on the progression of DR. Future research should assess whether IF can delay DR progression by comparing the proportions of non-proliferative and proliferative DR between IF and regular diet groups in datasets that incorporate standardized DR grading. Third, blood ketone level was not measured in this study. Therefore, it was difficult to evaluate the degree of ketosis by IF and determine the contribution of increased ketone levels from IF to the risk reduction in DR. Although not statistically significant, the proportion of participants with urine ketone levels of trace or higher was numerically higher in the IF group than the regular diet group (7.7% vs. 4.2%). This suggests a potential trend toward increased ketone production in the IF group. Future prospective studies incorporating direct biochemical assessments, such as blood β-hydroxybutyrate measurements, will be important to confirm the presence and degree of ketosis and to elucidate the potential mechanistic links between IF, ketone metabolism, and both the prevalence and severity of DR. Fourth, it was not feasible to distinguish the types of diabetes based on the data used in this study. Considering the pathophysiologic differences between type 1 and type 2 diabetes [43], future studies should account for diabetes subtype when evaluating the effects of IF. Of the total study participants, 5 individuals (0.5%) were insulin-only users. The minimum age at diagnosis among them was 28 years, and three were diagnosed with diabetes in their 20 s to 30 s, suggesting possible latent autoimmune diabetes in adults (LADA), within the type 1 diabetes spectrum [38,43]. Taken together, the proportion of participants with potential type 1 diabetes was less than 0.5%, indicating that the majority of the study population can be considered to have type 2 diabetes. Fifth, the participants had a relatively short reported duration of diabetes, and it remains uncertain whether the association between IF and reduced DR risk would be consistent across longer diabetes durations. In addition, differences in diabetes duration between the IF and regular diet groups may have introduced residual confounding. Although subgroup and sensitivity analyses using various diabetes duration cutoffs were performed to address this issue, diabetes duration was self-reported and may have underestimated the true duration of hyperglycemia, particularly in type 2 diabetes, where a prolonged asymptomatic period is common. Notably, previous large-scale epidemiologic studies have shown that a substantial proportion of patients, approximately 13–21%, already exhibit mild DR at the time of diagnosis or during the early stage of diabetes [44,45,46]. Therefore, the observed DR prevalence among participants with short reported diabetes duration should be interpreted with caution. Sixth, differences in lipid profiles and BMI between groups could independently influence DR risk. In previous studies, elevated triglyceride and LDL-cholesterol levels were associated with an increased risk of DR occurrence [47,48,49,50]. On the other hand, the role of BMI for DR development were inconclusive [51,52,53,54]. In this study, we adjusted for lipid profiles and BMI in the multivariable logistic regression, and LDL-cholesterol, triglyceride, and BMI were not significantly associated with increased or reduced DR risk. The findings remained consistent in the PSM model. BMI and lipid profiles did not interact with the association between IF and lower DR risk either. Seventh, there was an imbalance in sample sizes between the IF and regular diet groups. To address this issue, we performed a 1:1 PSM analysis, but the resulting matched sample size was relatively small. Due to the difficulty in achieving an equal distribution of sufficient participants between the comparison groups, the generalizability of findings may be limited.

## 5. Conclusions

In conclusion, IF was associated with a significantly reduced risk for DR compared with regular diet. The decreased risk of DR associated with IF did not show heterogeneity across subgroups. In sensitivity analyses stratified by diabetes duration, IF continued to show a lower risk of DR. In the 1:1 PSM model, the protective association of IF with DR remained consistent. Nevertheless, to overcome the limitations in causal inference inherent to the cross-sectional and retrospective design of the current study, large-scale prospective cohort studies or randomized controlled trials are required to validate the potential protective role of IF in the prevention of DR. Future studies should also assess how the impact of IF may differ at different stages of DR (non-proliferative and proliferative) and diabetes (prediabetes and diabetes) to better understand the potential benefits of IF in various clinical contexts. This approach will be useful for the effective implementation and recommendation of IF in real-world clinical practice.

## Figures and Tables

**Table 1:** Baseline Characteristics of Study Subjects.

	Regular Diet (*n* = 831)	Intermittent Fasting (*n* = 91)	*p*-Value
Age (years)	66.0 [58.0–74.0]	56.0 [48.0–62.5]	**<0.001**
Male (*n* (%))	425 (51.1%)	47 (51.6%)	>0.999
BMI (kg/m^2^)	24.8 [22.7–27.1]	25.8 [24.1–27.9]	**0.009**
Smoking ^a^ (*n* (%))	375 (45.1%)	46 (50.5%)	0.381
High-risk drinking ^b^ (*n* (%))	68 (8.2%)	5 (5.5%)	0.486
Comorbidities			
Hypertension (*n* (%))	548 (65.9%)	50 (54.9%)	**0.049**
Dyslipidemia (*n* (%))	457 (55.0%)	54 (59.3%)	0.496
Stroke (*n* (%))	51 (6.1%)	3 (3.3%)	0.390
Cardiovascular disease (*n* (%))	53 (6.5%)	7 (7.9%)	0.802
Diabetes-related characteristics			
Status of diabetes			0.590
Diabetes (*n* (%))	753 (90.6%)	84 (92.3%)	
Prediabetes (*n* (%))	78 (9.4%)	7 (7.7%)	
Diabetic retinopathy ^c^ (*n* (%))	171 (20.6%)	8 (8.8%)	**0.010**
Duration of diabetes (years)	3.0 [0.0–10.0]	1.0 [0.0–5.0]	**0.001**
Insulin use (*n* (%)) ^d^	35 (4.7%)	4 (4.4%)	>0.999
Biochemical profiles			
Fasting blood glucose (mg/dL)	130.0 [115.0–150.0]	133.0 [118.5–151.5]	0.217
HbA1c (%)	6.8 [6.4–7.5]	6.6 [6.3–7.3]	0.607
Total cholesterol (mg/dL)	168.0 [145.0–204.0]	189.0 [154.0–217.0]	**0.001**
Triglyceride (mg/dL)	133.0 [92.0–192.0]	153.0 [105.5–253.5]	**0.002**
HDL-cholesterol (mg/dL)	44.0 [38.0–52.0]	44.0 [38.9–52.0]	0.539
LDL-cholesterol (mg/dL)	93.6 [73.1–121.0]	106.8 [81.0–132.8]	**0.007**
BUN (mg/dL)	16.0 [14.0–20.0]	15.0 [13.0–18.0]	**0.036**
Creatinine (mg/dL)	0.8 [0.7–1.0]	0.8 [0.7–1.0]	0.942
AST (IU/L)	23.0 [19.0–29.0]	24.0 [18.0–32.0]	0.486
ALT (IU/L)	22.0 [16.0–31.0]	25.0 [17.0–42.0]	**0.023**
Urine ketone (≥ Trace) (*n*, (%))	35 (4.2%)	7 (7.7%)	0.212
Calorie intake			
Total calorie intake per day (Kcal)	1741.8 [1279.3–2293.7]	1597.5 [1191.8–1956.6]	0.057
Carbohydrate % of calorie	69.5 [60.3–76.4]	64.3 [51.6–74.2]	**0.002**
Protein % of calorie	13.2 [11.0–15.5]	13.3 [11.3–16.4]	0.415
Fat % of calorie	13.9 [9.2–19.2]	15.9 [11.2–24.2]	**0.003**

ALT, alanine aminotransferase; AST, aspartate aminotransferase; BMI, body mass index; BUN, blood urea nitrogen; HbA1c, glycated hemoglobin; HDL, high-density lipoprotein; LDL, low-density lipoprotein. ^a^ Smoking is defined as smoking over 100 cigarettes in his or her life. ^b^ High-risk drinking is defined as drinking over 7 cups for men and over 5 cups for women per day. ^c^ DR in this table indicates cases newly diagnosed through fundus photography during the survey year. ^d^ Insulin users include both insulin-only users and those using insulin with oral antidiabetic agents. Bold font in *p*-value indicates statistical significance.

## Data Availability

The original data presented in the study are openly available in KNHANES at https://knhanes.kdca.go.kr (continuously accessible).

## Supplementary Materials

The following supporting information can be downloaded at: https://www.mdpi.com/article/10.3390/nu18111696/s1, Supplementary Table S1: Baseline characteristics of study subjects before and after 1:1 PSM; Supplementary Table S2: Multivariable logistic regression analysis for association between diabetic retinopathy and intermittent fasting across models in a propensity score-matched cohort; Supplementary Table S3: Multiple logistic regression analyses to determine independent factors for the risks of diabetic retinopathy in a propensity score-matched cohort; Supplementary Table S4: Subgroup analyses of association between intermittent fasting and risk of diabetic retinopathy; Supplementary Table S5: Risk of diabetic retinopathy associated with intermittent fasting diet by duration of diabetes.

## Abbreviations

ALT	Alanine aminotransferase
AST	Aspartate aminotransferase
BMI	Body mass index
CI	Confidence interval
DR	Diabetic retinopathy
HbA1c	Glycated hemoglobin
HDL	High-density lipoprotein
IF	Intermittent fasting
KNHANES	Korean National Health and Nutrition Examination Survey
LDL	Low-density lipoprotein
OR	Odds ratio
SIRT1	Sirtuin 1
