# Supplementary material for: Intermittent Fasting and Risk of Diabetic Retinopathy: Retrospective Data from the National Health and Nutrition Examination Survey
Source: Nutrients. 2026 May 26;18(11):1696. doi: 10.3390/nu18111696 (PMC13258044; doi:10.3390/nu18111696)
Supplement: Supplementary file 1 [file nutrients-18-01696-s001.zip › nutrients-4327496-supplementary.pdf]

Supplementary Table S1. Baseline characteristics of study subjects before and after 1:1 PSM

	Before PSM				*After 1:1 PSM*			
	Total cohort (n = 922)	Control (regular diet) (n = 831)	Intermittent fasting (n = 91)	*P*-value	Total cohort (n = 172)	Control (regular diet) (n = 86)	Intermittent fasting (n = 86)	*P*-value
Age (years)	64.00 [56.00–73.00]	66.00 [58.00–74.00]	56.00 [48.00–62.50]	**<0.001**	55.00 [48.00–62.25]	54.00 [48.00–61.75]	56.00 [48.25–63.00]	0.377
Sex				0.999				0.762
Male	472 (51.2%)	425 (51.1%)	47 (51.6%)		89 (51.7%)	46 (53.5%)	43 (50.0%)	
Female	450 (48.8%)	406 (48.9%)	44 (48.4%)		83 (48.3%)	40 (46.5%)	43 (50.0%)	
BMI (kg/m^2)	24.87 [22.77–27.24]	24.80 [22.69–27.10]	25.77 [24.07–27.87]	**0.009**	26.19 [23.88–28.24]	26.43 [23.72–28.67]	25.83 [24.06–27.97]	0.536
Smoking				0.381				0.647
Ever smoker[a]	421 (45.7%)	375 (45.1%)	46 (50.5%)		88 (51.2%)	46 (53.5%)	42 (48.8%)	
Never smoker[b]	501 (54.3%)	456 (54.9%)	45 (49.5%)		84 (48.8%)	40 (46.5%)	44 (51.2%)	
Drinking				0.486				0.999
High-risk drinking[c]	73 (7.9%)	68 (8.2%)	5 (5.5%)		11 (6.4%)	6 (7.0%)	5 (5.8%)	
Non-high-risk drinking	849 (92.1%)	763 (91.8%)	86 (94.5%)		161 (93.6%)	80 (93.0%)	81 (94.2%)	
Medical comorbidities								
Hypertension (n, %)	598 (64.9%)	548 (65.9%)	50 (54.9%)	**0.049**	96 (55.8%)	48 (55.8%)	48 (55.8%)	0.999
Dyslipidemia (n, %)	511 (55.4%)	457 (55.0%)	54 (59.3%)	0.496	108 (62.8%)	56 (65.1%)	52 (60.5%)	0.636
Stroke (n, %)	54 (5.9%)	51 (6.1%)	3 (3.3%)	0.390	6 (3.5%)	3 (3.5%)	3 (3.5%)	0.765
Cardiovascular disease (n, %)	60 (6.7%)	53 (6.5%)	7 (7.9%)	0.802	12 (7.0%)	5 (5.8%)	7 (8.1%)	0.999
Status of diabetes				0.734				0.999
Diabetes	837 (90.8%)	753 (90.6%)	84 (92.3%)		157 (91.3%)	78 (90.7%)	79 (91.9%)	
Prediabetes	85 (9.2%)	78 (9.4%)	7 (7.7%)		15 (8.7%)	8 (9.3%)	7 (8.1%)	
Insulin use[d]				0.999				0.999
Yes	43 (4.7%)	39 (4.7%)	4 (4.4%)		7 (4.1%)	3 (3.5%)	4 (4.7%)	

No	879 (95.3%)	792 (95.3%)	87 (95.6%)		165 (95.9%)	83 (96.5%)	82 (95.3%)	
Diabetes duration (years)	3.00 [0.00–10.00]	3.00 [0.00–10.00]	1.00 [0.00–5.00]	**0.001**	1.00 [0.00–5.00]	1.50 [0.00–5.00]	1.00 [0.00–5.75]	0.860
Blood biochemical profiles								
Fasting blood glucose (mg/dL)	131.00 [115.00–150.00]	130.00 [115.00–150.00]	133.00 [118.50–151.50]	0.217	133.50 [118.75–154.00]	133.50 [120.00–153.25]	134.00 [118.25–153.50]	0.783
HbA1c (%)	6.80 [6.40–7.50]	6.80 [6.40–7.50]	6.60 [6.30–7.35]	0.607	6.80 [6.40–7.43]	6.85 [6.50–7.40]	6.75 [6.40–7.62]	0.790
Total cholesterol (mg/dL)	169.00 [146.00–206.00]	168.00 [145.00–204.00]	189.00 [154.00–217.00]	**0.001**	183.00 [153.00–220.25]	174.50 [153.25–219.25]	186.00 [153.50–220.00]	0.484
Triglyceride (mg/dL)	135.00 [94.00–194.00]	133.00 [92.00–192.00]	153.00 [105.50–253.50]	**0.002**	150.00 [100.75–232.50]	147.00 [98.25–210.50]	150.50 [104.50–249.00]	0.344
HDL-cholesterol (mg/dL)	44.00 [38.00–52.00]	44.00 [38.00–52.00]	44.00 [38.87–52.00]	0.539	45.16 [40.00–54.00]	45.66 [40.25–55.00]	45.00 [39.00–52.00]	0.412
LDL-cholesterol (mg/dL)	94.51 [74.00–123.00]	93.60 [73.10–121.05]	106.80 [81.00–132.80]	**0.007**	100.40 [79.35–130.35]	93.20 [77.84–125.20]	108.90 [83.03–134.65]	0.178
BUN (mg/dL)	16.00 [14.00–20.00]	16.00 [14.00–20.00]	15.00 [13.00–18.00]	**0.036**	15.00 [13.00–18.00]	15.00 [13.25–18.00]	15.00 [13.00–18.00]	0.639
Creatinine (mg/dL)	0.80 [0.68–0.96]	0.80 [0.68–0.96]	0.81 [0.67–0.96]	0.942	0.80 [0.67–0.94]	0.78 [0.68–0.93]	0.81 [0.67–0.96]	0.797
AST (IU/L)	23.00 [19.00–29.75]	23.00 [19.00–29.00]	24.00 [18.00–32.00]	0.486	23.00 [18.00–29.25]	23.00 [19.00–29.00]	24.00 [18.00–31.25]	0.577
ALT (IU/L)	22.00 [16.00–31.00]	22.00 [16.00–31.00]	25.00 [17.00–42.00]	**0.023**	25.00 [17.00–38.00]	25.00 [18.00–36.00]	24.50 [17.00–39.75]	0.861
Urine ketone (≥Trace) (n, (%))	42 (4.6)	35 (4.2)	7 (7.7)	0.212	16 (9.3)	9 (10.5)	7 (8.1)	0.793
Calorie intake								
Carbohydrate % of calorie	68.99 [59.99, 76.31]	69.49 [60.31, 76.40]	64.29 [51.61, 74.21]	**0.002**	64.96 [51.69, 73.92]	65.45 [52.04, 73.45]	64.59 [51.58, 75.04]	0.755
Protein % of calorie	13.19 [11.00, 15.54]	13.18 [10.98, 15.49]	13.33 [11.28, 16.44]	0.414	13.47 [11.68, 16.47]	13.96 [12.64, 16.64]	13.15 [11.08, 16.26]	0.066
Fat % of calorie	14.05 [9.41, 19.76]	13.89 [9.22, 19.17]	15.90 [11.25, 24.22]	**0.003**	16.46 [11.32, 23.89]	16.68 [12.11, 23.01]	15.93 [10.99, 24.23]	0.833

ALT, alanine aminotransferase; AST, aspartate aminotransferase; BMI, body mass index; BUN, blood urea nitrogen; HbA1c, glycated hemoglobin; HDL, high-density lipoprotein: LDL, low-density lipoprotein; PSM, propensity score matching.

[a] Ever smoker is defined as smoking over 100 cigarettes in lifetime.

[b] Never smoker is defined as smoking fewer than 100 cigarettes in his or her lifetime.

[c] High-risk drinking is defined as drinking over 7 cups for men and over 5 cups for women per day.

[d] Insulin users include both insulin-only users and those using insulin with oral antidiabetic agents.

1:1 PSM was done to balance baseline characteristics (all variables in Supplementary Table S1 except diabetes duration).

Bold font in p-value indicates statistical significance ($p < 0.05$).

Supplementary Table S2. Multivariable logistic regression analysis for association between diabetic retinopathy and intermittent fasting across models in a propensity score-matched cohort

Groups	Regular diet Odds ratio	Intermittent fasting Odds ratio (95% CI)	*P*-value
Model 1†	1.00‖	0.338 (0.133–0.793)	**0.016**
Model 2‡	1.00‖	0.327 (0.126–0.776)	**0.015**
Model 3§	1.00‖	0.291 (0.105–0.734)	**0.012**
Model 4¶	1.00‖	0.184 (0.053–0.536)	**0.004**
Model 5#	1.00‖	0.135 (0.034–0.430)	**0.001**

CI, confidence interval.

1:1 PSM was done to balance baseline characteristics (all variables in Supplementary Table S1 except diabetes duration).

Model 1†: Unadjusted.

Model 2‡: Adjusted for age, sex, and BMI.

Model 3§: Adjusted for model 2 covariates plus hypertension, duration of diabetes, diabetes vs. prediabetes, and smoking.

Model 4¶: Adjusted for model 3 covariates plus insulin use, fasting glucose, HbA1c, triglyceride, LDL-cholesterol, and ALT.

Model 5#: Adjusted for model 4 covariates plus total calories intake per day and proportions of macronutrients; carbohydrate, protein, and fat.

‖ Reference category.

Bold font in p-value indicates statistical significance ($p < 0.05$).

Supplementary Table S3. Multiple logistic regression analyses to determine independent factors for the risks of diabetic retinopathy in a propensity score-matched cohort

	Diabetic retinopathy	
	Odds ratio (95% CI)	*P*-value
Intermittent fasting (vs. regular diet)	0.135 (0.034–0.430)	**0.001**
Age (years)	1.004 (0.939–1.073)	0.901
Male (vs. female)	0.499 (0.092–2.474)	0.404
BMI (kg/m^2)	0.914 (0.777–1.065)	0.256
Hypertension	0.960 (0.322–2.923)	0.942
Diabetes duration (years)	1.149 (1.053–1.271)	**0.003**
Smoking (yes vs. no)[a]	1.228 (0.246–6.039)	0.800
Insulin use (yes vs. no)	1.247 (0.091–14.319)	0.861
Fasting blood glucose (mg/dL)	1.019 (0.999–1.041)	0.060
HbA1c (%)	1.102 (0.608–2.010)	0.746
Triglyceride (mg/dL)	1.000 (0.997–1.003)	0.892
LDL-cholesterol (mg/dL)	1.007 (0.993–1.021)	0.332
ALT(IU/L)	0.998 (0.967–1.025)	0.900
Total calories intake per day (Kcal)	1.000 (0.999–1.001)	0.740

ALT, alanine aminotransferase; BMI, body mass index; CI, confidence interval; HbA1c, glycated hemoglobin; LDL, low-density lipoprotein.

[a] Smoking was defined as having consumed over 100 cigarettes in one's lifetime.

1:1 PSM was done to balance baseline characteristics (all variables in Supplementary Table S1 except diabetes duration). Additional adjustment was performed in accordance with Model 5 in Supplementary Table S2.

Bold font in p-value indicates statistical significance ($p < 0.05$).

Supplementary Table S4. Subgroup analyses of association between intermittent fasting and risk of diabetic retinopathy

Variable	No. of patients	OR (95% CI)	*P*-value	*P* for interaction
Age (year)				0.235
< 60	315	0.48 (0.16–1.28)	0.169	
≥ 60	607	0.08 (0.01–0.44)	**0.020**	
Sex				0.158
Male	472	0.50 (0.17–1.29)	0.179	
Female	450	0.15 (0.02–0.59)	**0.021**	
BMI (kg/m^2)				0.534
< 23	255	0.10 (0.04–0.71)	0.052	
≥ 23	667	0.33 (0.12–0.76)	**0.015**	
Hypertension				0.828
No	324	0.23 (0.05–0.80)	**0.038**	
Yes	598	0.35 (0.11–0.93)	0.051	
Diabetes duration (year)				0.965
< 3	432	0.26 (0.06–0.84)	**0.045**	
≥ 3	490	0.28 (0.08–0.79)	**0.026**	
Smoking[a]				0.167
No	501	0.12 (0.02–0.50)	**0.011**	
Yes	421	0.55 (0.18–1.46)	0.258	
Insulin use				0.693
No	879	0.31 (0.12–0.70)	**0.009**	
Yes	43	0.0004 (0.0001–6.31)	0.692	
Fasting blood glucose (mg/dL)				0.076
< 126	354	0.11 (0.01-0.78)	0.068	
≥ 126	568	0.33 (0.12-0.77)	**0.017**	
HbA1c (%)				0.350
< 6.5	253	0.46 (0.06–2.05)	0.370	
≥ 6.5	669	0.25 (0.08–0.61)	**0.005**	
Triglyceride (mg/dL)				0.232
< 150	532	0.12 (0.02–0.47)	**0.008**	
≥ 150	390	0.50 (0.16–1.35)	0.199	
LDL-cholesterol (mg/dL)				0.582
< 100	521	0.22 (0.05–0.69)	**0.022**	
≥ 100	401	0.46 (0.13–1.32)	0.180	
ALT (IU/L)[c]				0.214
Elevated ALT	647	0.27 (0.08–0.71)	**0.014**	
Normal ALT	275	0.51 (0.10–1.84)	0.346	
Overeating[b]				0.225
No	463	0.20 (0.05–0.60)	**0.009**	
Yes	454	0.59 (0.14–1.95)	0.422	
Proportion of carbohydrate (%)				0.263
< 45	74	1.06 (0.05–17.38)	0.969	
≥ 45	848	0.23 (0.08–0.56)	**0.003**	

Proportion of fat (%)				0.944
< 25	809	0.34 (0.13–0.78)	**0.018**	
≥ 25	113	0.15 (0.01–1.56)	0.188	

ALT, alanine aminotransferase; BMI, body mass index; CI, confidence interval; HbA1c, glycated hemoglobin; LDL, low-density lipoprotein; OR, odds ratio.

[a] Smoking is defined as smoking over 100 cigarettes in his or her life.

[b] Overeat is defined as total daily caloric intake of more than 1800 kcal.

[c] Elevated is defined as ALT, ALT > 33 IU/L in male and > 25 IU/L in female.

All models were adjusted for age, sex, BMI, hypertension, duration of diabetes, smoking, insulin use, fasting glucose, HbA1c, triglyceride, LDL-cholesterol, ALT, total calories intake per day, and proportions of macronutrients; carbohydrate, protein, and fat.

Regular diet group was used as the reference category.

Bold font in p-value indicates statistical significance ($p < 0.05$).

Supplementary Table S5. Risk of diabetic retinopathy associated with intermittent fasting diet by duration of diabetes

Groups	Odds ratio	95% confidence interval		*P*-value
		Lower limit	Upper limit	
Diabetes duration ≤ 3 yrs				
Model 1†	0.45	0.13	1.15	0.136
Model 2‡	0.35	0.10	0.92	0.051
Model 3§	0.33	0.10	0.89	**0.048**
Model 4¶	0.30	0.08	0.86	**0.043**
Model 5#	0.26	0.07	0.77	**0.027**
Diabetes duration ≤ 5 yrs				
Model 1†	0.41	0.12	1.04	0.095
Model 2‡	0.33	0.10	0.85	**0.041**
Model 3§	0.34	0.10	0.92	0.058
Model 4¶	0.33	0.09	0.92	0.055
Model 5#	0.30	0.08	0.83	**0.037**

Model 1†: Unadjusted.

Model 2‡: Adjusted for age, sex, and BMI.

Model 3§: Adjusted for model 2 covariates plus hypertension, duration of diabetes, smoking.

Model 4¶: Adjusted for model 3 covariates plus insulin use, fasting glucose, HbA1c, triglyceride, LDL-cholesterol, and ALT.

Model 5#: Adjusted for model 4 covariates plus total calories intake per day and proportions of macronutrients; carbohydrate, protein, and fat.

For individuals with prediabetes, diabetes duration was defined as 0 years.

The odds ratio was calculated using the regular diet group as the reference.

Bold font in p-value indicates statistical significance ($p < 0.05$).
